# Evaluation of Wild Lentil Species as Genetic Resources to Improve Drought Tolerance in Cultivated Lentil

**DOI:** 10.3389/fpls.2017.01129

**Published:** 2017-06-29

**Authors:** Linda Y. Gorim, Albert Vandenberg

**Affiliations:** Pulse Group, Department of Plant Sciences, College of Agriculture and Bioresources, University of SaskatchewanSaskatoon, SK, Canada

**Keywords:** wild lentil genotypes, soil horizons, root traits, drought strategies, growth parameters

## Abstract

Increasingly unpredictable annual rainfall amounts and distribution patterns have far reaching implications for pulse crop biology. Seedling and whole plant survival will be affected given that water is a key factor in plant photosynthesis and also influences the evolving disease spectrum that affects crops. The wild relatives of cultivated lentil are native to drought prone areas, making them good candidates for the evaluation of drought tolerance traits. We evaluated root and shoot traits of genotypes of cultivated lentil and five wild species grown under two water deficit regimes as well as fully watered conditions over a 13 week period indoors. Plants were grown in sectioned polyvinyl chloride (PVC) tubes containing field soil from the A, B, and C horizons. We found that root distribution into different soil horizons varied among wild lentil genotypes. Secondly, wild lentil genotypes employed diverse strategies such as delayed flowering, reduced transpiration rates, reduced plant height, and deep root systems to either escape, evade or tolerate drought conditions. In some cases, more than one drought strategy was observed within the same genotype. Sequence based classification of wild and cultivated genotypes did not explain patterns of drought response. The environmental conditions at their centers of origin may explain the patterns of drought strategies observed in wild lentils. The production of numerous small seeds by wild lentil genotypes may have implications for yield improvement in lentil breeding programs.

## Introduction

Lentil (*Lens culinaris* Medik.) is a high value indeterminate annual food legume crop with seeds that are rich in protein, iron, and zinc. It co-evolved in cropping systems that included wheat and barley and is produced mostly in marginal areas under rainfed conditions with a global average yield of 850 kg/ha (Erskine et al., 2011). Because lentils cook quickly, global consumption is rising faster than human population growth, and production is increasing in regions including Australia and the northern plains of North America. Similar to many legume crops, lentil is susceptible to abiotic stresses such as cold, drought, heat, salinity, soil nutrient deficiencies, and toxicities; drought and heat are considered its most important stresses worldwide (Turner et al., 2001). Climate forecasts suggest that variable annual rainfall patterns threaten the sustainability of lentil production by increasing the frequency of drought periods during the cropping season (Dai, 2011). Crop improvement efforts must be directed toward strategies that will allow production to be maintained during stress periods, especially those due to drought (Erskine et al., 2011). A short-term approach is to assess the available genetic diversity for drought tolerance in existing cultivated lentil germplasm. A longer-term approach is to use traits that may exist in and be transferable from wild crop relatives. In the case of lentil, its wild crop relatives originate from areas with variable climatic conditions and soils, and might have evolved to acquire a wide range of drought tolerance mechanisms. Cultivated lentil is reported to have two centers of origin, India and the Middle East (Ladizinsky, 1993), whereas wild lentil species may have multiple centers of origin (Pratap et al., 2014; Singh et al., 2014). The latest classification of the six wild lentil species on the basis of genotyping-by-sequencing (Wong et al., 2015) grouped the wild lentil species into four gene pools, of which five species can be used to make hybrids with cultivated lentil. Origins reported for wild lentils, with geographical coordinates, indicate they were collected mostly from Turkey but also from other locations such as Syria and Spain (Table 1).

**Table 1:** Cultivated and five wild lentil species, their abbreviated names, gene pool classifications, centers of origin, and the ecological conditions at their centers of origin.

***Lens* species and genotype**	**Abbreviated name**	**Gene pool**	**Centre of origin**	**Ecological conditions at centers of origin**
*culinaris* (Medik.) Eston	*L. cul*. Eston	Cultivated and Primary	Middle East	Vast ecological span (Ladizinsky, 1993). Mediterranean, semi-arid temperate, and sub-tropical savannah
*orientalis* (Boiss.) PI 572376	*L. ori*. PI 572376	Primary	Latitude: 37.67 Longitude: 29.13 (Denizli, SW Turkey)	Mountainous region with hot summers and subzero winters; Calcareous loam soils; Annual precipitation about 550 mm (Yaldiz et al., 2005).
*orientalis* (Boiss.) IG 72643	*L. ori*. IG 72643	Primary	Latitude: 36.3375 Longitude: 36.8389. (Reyhan / Aleppo, Syria)	Hot summers and winters with spells below zero. Soils are inceptisols. Annual precipitation is between 200 and 250 mm (FAO, 2016).
*tomentosus* (Ladiz.) IG 72805	*L. tom*. IG 72805	Primary	Latitude: 37.75 Longitude: 39.7667 (Sanliurfa, SE Turkey)	Extremely dry hot summers, cool moist winters with frost events and sporadic snowfall. Soils are calcisols and vertisols. Annual precipitation of about 410 mm (Mehmet et al., 2005).
*odemensis* (Ladiz.) IG 72623	*L. ode*. IG 72623	Secondary	Latitude: 37.44 Longitude: 41.0167 (Mardin, SE Turkey)	Mediterranean climate with hot summers and cold, wet, and occasionally snowy winters. Soils are cambisols. Annual precipitation between 428 and 640 mm (Sayar and Han, 2016).
*lamottei* (Czefr.) IG 110813	*L. lam*. IG 110813	Secondary	Latitude: 37.4167 Longitude: −4.25 (Xativa, Valencia, Spain)	Hot semi-arid climate, mild winters, long hot summers (Duran et al., 2013). Soils are leptosols and luvisols. Highly variable rainfall with annual precipitation of about 475 mm (Cerda and Doerr, 2005).
*ervoides* (Brign.) IG 72815	*L. erv*. IG 72815	Tertiary	Latitude: 37.6 Longitude: 36.5 (Kahramammaras, SE Turkey)	Temperate Mediterranean climate with hot dry summers and cold winters. Soils are calcisols and vertisols. Annual precipitation od about 730 mm (Doygun et al., 2008).
*ervoides* (Brign.) L-01-827A	*L. erv*. L-01-827A	Tertiary	Selection from ICARDA accession (see Fiala et al., 2009)	Assumed to be the same as *L. erv*. IG 72815. Species has a wide distribution.

*Note: Gene pool classifications and all geographical coordinates obtained from Wong et al. (2015)*.

Previous investigations of drought tolerance characteristics of wild lentil by Pratap et al. (2014) and Singh et al. (2014) focused on above ground agro-morphological traits. Published research on assessment of wild genotypes from the perspective of root growth is very limited, especially comparisons of growth under drought vs. well-watered conditions. Aside from providing mechanical support of the above-ground plant parts, roots are the major organ for acquiring nutrients and water from the surrounding soil (Chen et al., 2015). Furthermore, the identification of relevant root traits offers the potential to increase grain yield in plants both under conditions of poor soil resources and under optimal soil water and nutrient supply conditions (Gahoonia et al., 2005; Chen et al., 2015). Above ground, lentil plants employ other mechanisms to evade water deficits. Examples include the development of pubescence on plant parts to prevent overheating, and early or delayed flowering to evade or escape water stress. Another example is the ability to tolerate extreme stress by accumulating products of photosynthesis, leading to high osmotic concentrations in leaf cells under conditions of water stress (Turner et al., 2001). No systematic studies have identified what mechanisms allow wild lentil species to flourish in environments with variable amounts of rainfall. We hypothesized that wild lentils classified in the same gene pools (Wong et al., 2015) would have similar drought tolerance under moisture deficit conditions and that the mechanisms they employ to survive drought conditions are different from those in cultivated genotypes. Our second hypothesis is that root growth parameters are different in the different wild lentil species because they are found in such a wide natural range of climatic and soil conditions. Our overall goal was to identify the different drought mechanisms in wild lentil genotypes across species by assessing both above ground plant characteristics and their root systems.

## Materials and methods

### Soil materials

The experiment was carried out in the controlled environment facility at the College of Agriculture and Bioresources at the University of Saskatchewan, Canada (lat. 52.133; long. −106.631). Soil was collected from an uncultivated corner of the Saskatchewan Pulse Growers (SPG) farm located east of Saskatoon (coordinates: 52.070908, −106.443905), Canada in August 2015. The soil was classified as an orthic dark brown chernozem with the A and B horizons both described as loam and the C horizon as clay loam. Soils from the A, B, and C horizons were removed separately. The soil pH ranged from 7.6 in the A horizon to 9.0 in the C horizon. The soil had extremely low nitrogen (2.24 kg ha^−1^) in the A and B horizons, slightly higher nitrogen (3.36 kg ha^−1^) in the C horizon, sufficient potassium, very low phosphorus, and sufficient micronutrients.

The soil was placed into 10 cm diameter × 60 cm length tubes that were divided into three 20-cm sub-sections corresponding to the depths of the A, B, and C horizons observed in the field. The top section of each tube was filled with 1.8 kg of soil from the A horizon to allow sufficient space for watering. The middle and bottom tube sections were filled with 2 kg of soil from the B and C horizons, respectively. The bottom section of the tube was sealed with a fine mesh to provide drainage and prevent soil loss. The amount of soil in each tube sub-section was determined by first measuring the moisture content of each load of soil using a Sartorius MA30 moisture analyzer (MA30 Sartorius Corp. NY, USA) and then adjusting for water content. The A, B, and C horizons were held together by duct tape. Ten random 60-cm filled tubes were then filled with water to saturation, their tops covered with aluminum foil, and settling allowed for 48 h until no water was observed to leave the base of the tube. The amount of water held by each tube was then calculated by subtracting the weight of both the empty tube and dry soil, and this was referred to as the amount of water at 100% field capacity (FC). The amount of water required to achieve both 40 and 25% FC was then determined. Fully watered tubes were maintained at 80% FC to avoid flooding. Before sowing, each tube received an application of 200 mL of modified Hoagland solution that included calcium chloride (60.5 mM), micronutrients (12.1 mM), FeEDTA (12.1 mM), potassium hydrogen phosphate (12.1 mM), and magnesium sulfate (4.1 mM).

### Plant materials

Table 1 details the names, abbreviations, centers of origin, and ecological conditions at the centers of origin for the cultivated and five wild lentil species used in the study. Seeds of wild and cultivated species were scarified, washed in bleach, and then pre-germinated in a dark chamber at 22°C. After 3 days, seedlings with radicle length >2 cm were selected and transplanted into soil tubes. Rhizobium inoculum (*Rhizobium leguminosarum* biovar *viceae* strain 1435; Nodulator XL SCG, Becker Underwood, Canada) was added next to transplanted seedlings at a rate of 6 g per tube.

**Figure 1:**
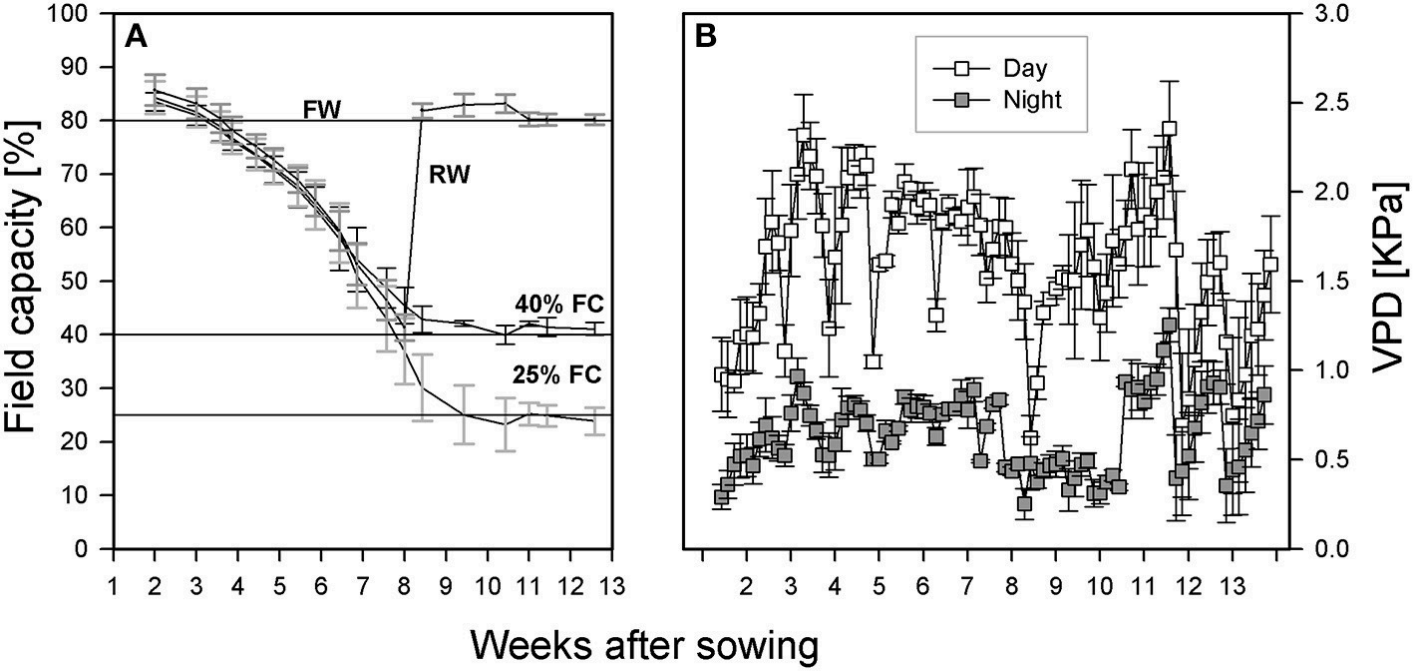
**(A)** Growth conditions under which all lentil genotypes were grown (FW, path for plants grown and maintained under fully watered conditions; RW, path for plants whose tube moisture level was allowed to decline to 40% of field capacity (FC) before re-watering to FW; 40 and 25% FC: path for those plants whose tube moisture levels were allowed to decline to 40 and 25% of FC, respectively, and maintained at that level for the rest of the experiment). **(B)** Vapor pressure deficit (VPD) in the room throughout the growth period.

The experiment was a complete randomized block design with four replicates, four treatments, eight genotypes, 12 evaporation tubes as controls, and two assessments were carried out at pod filling and at harvest, resulting in a total of 272 tubes. Fully watered treatments (Figure 1A) featured tubes maintained at 80% FC throughout the experiment. Two sets of four tubes were left un-watered until 40% FC, then one set was re-watered and maintained at 80% FC (re-watered treatment). The other set was maintained at 40% FC (moderate drought). A final set of tubes was left to dry down to 25% and then maintained at that level (severe drought). The moisture level in each tube was maintained by weighing, recording the value, and topping up with water as needed every other day throughout the experiment. Four tubes without plants at each treatment level were used to estimate evaporation rates. The temperature was set to 21°C day/15°C night with a day length of 16 h. Light intensity ranged from 308 to 392 μmol·m^−2^·s^−1^ depending on tube position and plant height. The light bulbs in the room were of two types (T-5 Florescence bulb # 835 Philips, ON, Canada and LED light bars, 730 mm Far Red, Fluence Bioengineering, Austin, TX, USA). Tube positions within each block were re-randomized at each weighing throughout the experiment to minimize light position effects. Temperature and humidity loggers (ibutton DS1923, Embedded Data Systems, Lawrenceburg, KY, USA) were placed in random pots in each block to monitor environmental conditions. These data were used to calculate vapor pressure deficit as described by Allen et al. (1998) and presented in Figure 1B.

### Parameters evaluated

Half of the plants were evaluated for growth parameters at 11 weeks after sowing (WAS) while the other half were left to flower and set seeds. Days to flowering (DTF) was recorded and plant height was measured from the soil level in tubes to the tip of the uppermost leaf. Four replicates of each genotype were harvested. The above ground plant material was placed in paper bags, oven dried at 70°C for 48 h, and then weighed to estimate biomass. Vertical growth rates were estimated by calculating relative growth rate (RGR) using plant height at 6 and 13 WAS calculate RGR by applying the following formula:

$$\begin{array}{lll} \text{RGR} & = & \left(\text{height at }13\text{ WAS}-\text{height at }6\text{ WAS}\right)/\\ \text{ time difference between measurements in weeks}. \end{array}$$

Roots from each horizon in each tube were prepared for measurement using the following procedure. After cutting through the duct tape and root cylinder with a sharp knife, roots were collected on a 0.5 mm mesh screen to minimize root loss prior to washing and placement in Ziploc® bags. Debris and dead roots from the field soil were manually removed prior to root analysis with imaging software (WinRHIZO™ 2013, Regent Instruments Inc., Ste. Foy, Canada). Fresh root morphological traits estimated included root length, root surface area, root length per unit volume of soil (referred to as root length density), root volume, and root/shoot ratio (RSR; Liu et al., 2010). After analysis, the roots were placed in labeled paper bags, oven dried at 70°C for 48 h, and weighed prior to calculation of dry matter RSR. Transpiration rate for each treatment was calculated by subtracting the amount of water that evaporated from the tubes without plants from those with plants, and dividing this value by the number of days between weight measurements. Weighing and recording tube weights every other day throughout the experiment enabled estimation of the total amount of water transpired from 1 to 13 WAS. The sum of water used was divided by the total volume of root produced by each genotype and expressed as the total amount of water transpired per unit volume of root. The other half of the plants left to produce seeds were bagged in white mesh bags to capture shattering seeds. When more than 80% of the seeds on the plants matured, the number of pods on each plant was counted, and the number of seeds per plant and thousand seed weight recorded.

Means were compared among genotypes for above ground biomass, relative growth rate, and RSR. Least significant difference between means was calculated using the PROC GLM procedure in Statistical Analysis System software (SAS 9.4, SAS Institute, Cary, NC, USA). An analysis of variance was performed using PROC GLM to test for effects. Root parameter interactions were analyzed by employing the PROC MIXED procedure. Results are arranged phylogenetically so that the first four genotypes belong to the primary gene pool, followed by those in the secondary and tertiary gene pools, in accordance with the most recent classification (Wong et al., 2015).

## Results

### Effect of moisture level on plant growth and transpiration

Genotypes responded differently to drought treatments, and drought severity also triggered different responses irrespective of gene pool. *Lens cul*. Eston grown at 25% of FC flowered earlier than when it was grown at 40% of FC (Table 2). Wild lentil response to drought was variable. Drought induced delayed flowering in *L. ori*. IG 72623 but did not influence flowering time in *L. ori*. PI 572376 or *L. tom*. IG 72805. For genotypes in the secondary gene pool, DTF was significantly increased in *L. ode*. IG 72623 under moisture deficit conditions; flowering was also delayed but to a lesser extent in *L. lam*. IG 110813. DTF for *L. erv*. L-01-827A only increased when grown at 25% FC, but for *L. erv*. IG 72815 DTF increased for both drought levels (Table 2). Plant height, number of pods, and seed yield were reduced under water deficit conditions and in both *L. ode*. IG 72623 and *L. erv*. IG 72815, and no seeds were produced under 25% FC (Table 2). However, the tallest plants and highest number of seeds produced were observed in plants grown under fully watered conditions.

**Table 2:** Variation in quantitative traits in eight cultivated and wild lentil genotypes grown in 60-cm tubes at three different soil moisture levels.

**Traits traitcharacter**	***L. culinaris*** **Eston**			***L. orientalis*** **PI 572376**			***L. orientalis*** **IG 72643**			***L. tomentosus*** **IG 72805**		
	**FW**	**40% FC**	**25% FC**	**FW**	**40% FC**	**25% FC**	**FW**	**40% FC**	**25% FC**	**FW**	**40% FC**	**25% FC**
Days to >90% flowering	42	42	36	42	42	42	36	42	42	36	36	36
Plant height (cm)	42.9 (±1.7)	37.5 (±1.6)	36.2 (±0.5)	25.0 (± 1.8)	21.8 (±1.6)	19.0 (±0.6)	41.1 (±2.8)	30.4 (±0.4)	27.8 (±2.7)	40.3 (±2.3)	30.2 (±1.4)	28.3 (±0.6)
No. of pods plant^−1^	202.5 (±14.5)	68.3 (±13.8)	35.3 (±4.3)	188.5 (±42.0)	103.3 (±12.3)	61.5 (±6.4)	173.0 (±27.0)	54.5 (±5.2)	55.5 (±10.4)	306.8 (±33.1)	104.8 (±15.0)	61.0 (± 4.3)
Seed yield (g plant^−1^)	7.2 (±0.3)	2.4 (±0.5)	1.3 (±0.1)	4.8 (±0.2)	1.4 (±0.1)	0.3 (±0.1)	4.6 (±0.4)	1.0 (±0.4)	0.9 (±0.6)	4.6 (±0.2)	1.2 (±0.5)	0.6 (±0.1)
1000 SDWT (g)	35.0 (±0.4)	33.9 (±1.0)	28.9 (±2.1)	7.7 (±0.1)	8.3 (±0.3)	8.4 (±0.3)	16.5 (±0.4)	16.0 (±0.1)	13.0 (± 1.8)	13.0 (±0.3)	11.0 (±1.6)	11.2 (±0.2)
	***L. odemensis*** **IG 72623**			***L. lamottei*** **IG 110813**			***L. ervoides*** **L-01-827A**			***L. ervoides*** **IG 72815**		
Days to >90% flowering	42	74	74	36	42	42	28	28	36	60	74	74
Plant height (cm)	43.8 (±3.6)	36.8 (±2.4)	36.9 (±1.1)	39.2 (± 3.5)	37.9 (±2.8)	33.9 (±1.0)	39.8 (±2.9)	30.5 (±1.5)	32.2 (±0.9)	46.2 (±0.5)	33.5 (±0.7)	31.4 (±2.0)
No. of pods plant^−1^	57.5 (±8.8)	28.0 (±8.9)	NS	178.7 (± 12.0)	61.3 (±10.7)	59.3 (±5.8)	240.0 (±32.5)	83.3 (±20.3)	70.5 (±9.8)	41.0 (±13.7)	24.0 (±7.4)	NS
Seed yield (g plant^−1^)	3.3 (±0.1)	0.8 (±0.2)	NS	2.5 (±0.7)	0.9 (±0.2)	0.9 (±0.3)	3.6 (±1.2)	1.2 (±0.2)	0.7 (±0.1)	2.0 (±0.2)	0.9 (±0.1)	NS
1,000 SDWT (g)	15.7 (±0.5)	14.4 (±0.7)	NS	15.6 (±0.2)	14.1 (±0.6)	12.8 (±0.9)	6.6 (±0.1)	6.7 (±0.1)	6.2 (±0.1)	7.4 (±0.2)	7.1 (±0.1)	NS

*FW, Fully watered plants maintained under fully watered conditions; 40 and 25% FC, tube moisture levels were allowed to decline to 40 or 25%, respectively, of field capacity (FC) and then maintained at that level for the rest of the experiment; SDWT, seed weight; NS, no seed produce*.

**Figure 2:**
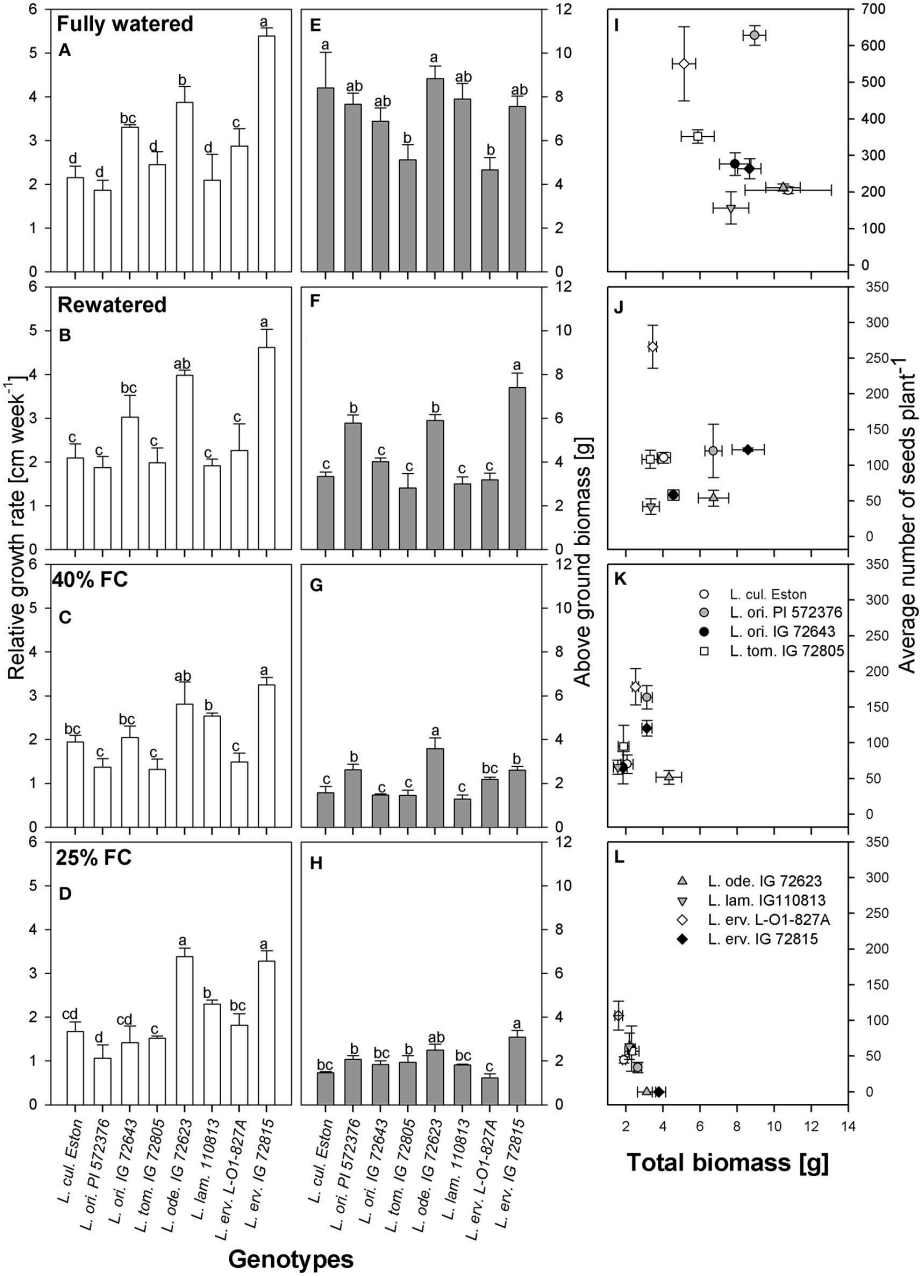
Comparisons of relative growth rate **(A–D)**, above ground biomass **(E–H)**, and the relationship between total biomass and number of seeds produced **(I–L)** between cultivated and wild lentil genotypes and within wild lentil genotypes that were grown under four conditions: fully watered, allowed to dry to 40% of field capacity (FC) and then re-watered, and allowed to dry to 40 or 25% FC and maintained at that level. Different lower case numbers denote significant differences at α = 5% at a given moisture level.

Vertical growth estimated using plant height was used to calculate RGR. Under fully watered conditions, *L. erv*. IG 72815 had the highest RGR and the tallest plants, followed by *L. ode*. IG 72623 and *L. ori*. IG 72643; the growth rates of these wild genotypes were significantly different (α = 5%) from those of the cultivated genotype, *L. cul*. Eston (Figure 2A). When plants were re-watered, the differences in RGR observed under fully watered conditions were still apparent (Figure 2B). However, when the plants were not re-watered but maintained at 40% FC, *L. ode*. IG 72623 and *L. erv*. IG 72815 had similar RGR but the RGR of *L. erv*. IG 72815 was significantly different from those of the other genotypes (Figure 2C). At 25% FC, genotypes belonging to the primary gene pool had significantly lower RGR than those in the secondary and tertiary gene pools except for *L. erv*. L-01-827A (Figure 2D). Interestingly, *L. ode*. IG 72623 and *L. erv*. IG 72815 had similar RGRs despite belonging to the secondary and tertiary gene pools, respectively. *L. lam*. 110813 and *L. erv*. L-01-827A also had similar RGR patterns. (Figure 2D).

Above ground biomass was measured to assess vertical growth resulting from branching. Under fully watered conditions, *L. tom*. IG 72805 and *L. erv*. L-01-827A had similar amount of biomass that were significantly lower (α = 5%) than those of cultivated lentil (Figure 2E). When plants were re-watered, significantly more biomass was produced by *L. erv*. IG 72815 compared to all other genotypes (Figure 2F). *L. ori*. PI 572376 and *L. ode*. IG 72623 had the next highest, producing biomass that was significantly greater than the remaining genotypes (Figure 2F). For plants that were not re-watered but maintained at 40% FC, however, *L. ode*. IG 72623 produced the highest amount of above ground biomass, followed by *L. ori*. PI 572376 and *L. erv*. IG 72815, with the remaining genotypes producing similar amounts (Figure 2G). When plants were exposed to severe drought, i.e., 25% FC, the amounts of biomass produced by *L. erv*. IG 72815 was similar to that of *L. ode*. IG 72623 and significantly higher than that produced by other genotypes. The least amount of biomass was produced by *L. erv*. L-01-827A (Figure 2H).

Resource allocation into either seed or biomass production was assessed by plotting the total amount of biomass produced, excluding seed biomass, against the total number of seeds under all moisture regimes (Figures 2I–L). High amounts of total biomass did not directly translate into yield for most genotypes, especially for *L. erv*. IG 72815. Under most conditions except fully watered, *L. erv*. L-01-827A produced the most seeds but the least total biomass. *L. ori*. PI 572376 was short and produced many branches that produced the most seeds under fully watered conditions (Figure 2I). *L. ori*. IG 72643 and *L. tom*. IG 72805 both produced an intermediate number of seeds under fully watered conditions that was significantly reduced under drought conditions. *L. ode*. IG 72623 produced the lowest number of seeds but had high biomass under both severe and moderate drought. When re-watered, this genotype produced more biomass rather than seeds (Figures 2J,K). Both *L. ode*. IG 72623 and *L. erv*. IG 72815 produced no seeds under severe drought.

**Figure 3:**
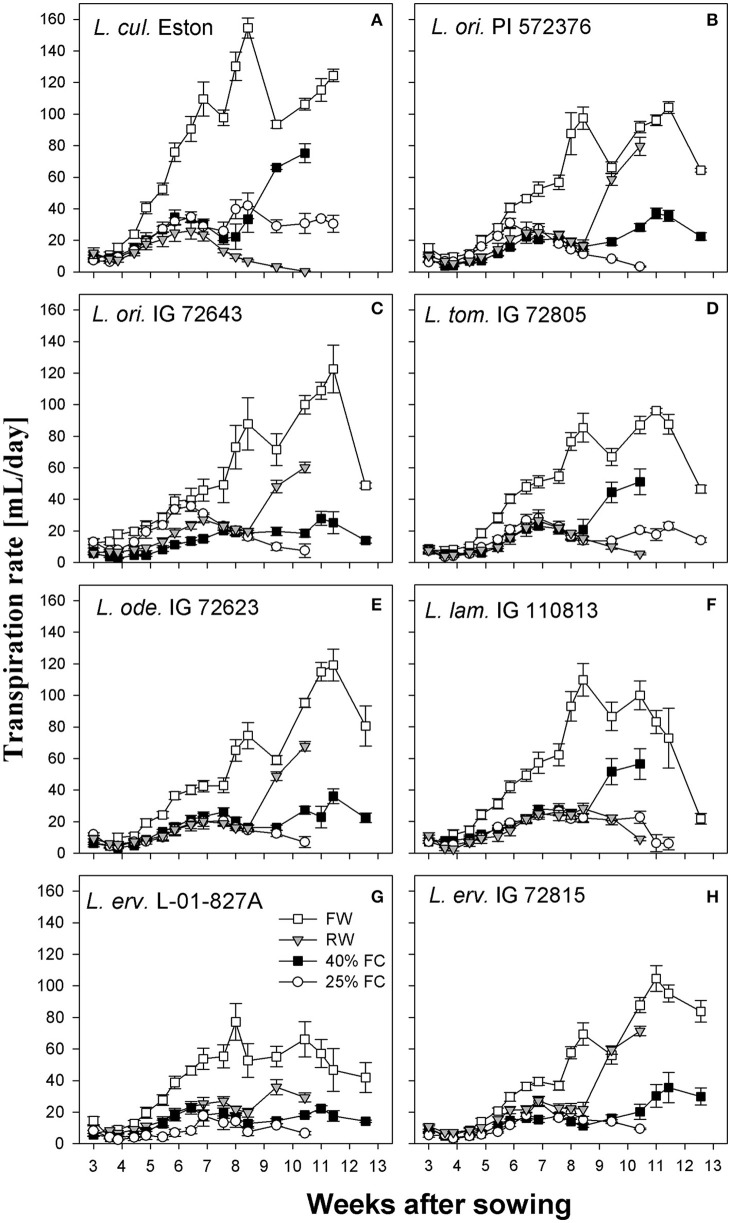
Transpiration rate of cultivated and wild lentil genotypes grown under different soil moisture levels. Plants were grown under four conditions: fully watered (FW), allowed to dry to 40% of field capacity (FC) and then re-watered (RW), and allowed to dry to 40 or 25% FC and maintained at that level.

Transpiration rates were higher under fully watered conditions in all genotypes, with a peak at around 8 WAS and a second peak at different intervals thereafter; however, the amount of water transpired varied among genotypes. Also, when plants were grown under stress conditions, transpiration rates were reduced irrespective of the stress severity especially during the first 8 WAS (Figure 3). *Lens culinaris* Eston transpired the most water (155 mL per day) at 8 WAS; this was its second peak, with an earlier peak between 6 and 7 WAS representing transpiration of 110 mL per day. Among wild lentil genotypes, *L. tom*. IG 72805 transpired the least amount of water under fully watered conditions, followed by *L. erv*. L-01-827A.

### Effects of moisture level on root growth parameters

**Figure 4:**
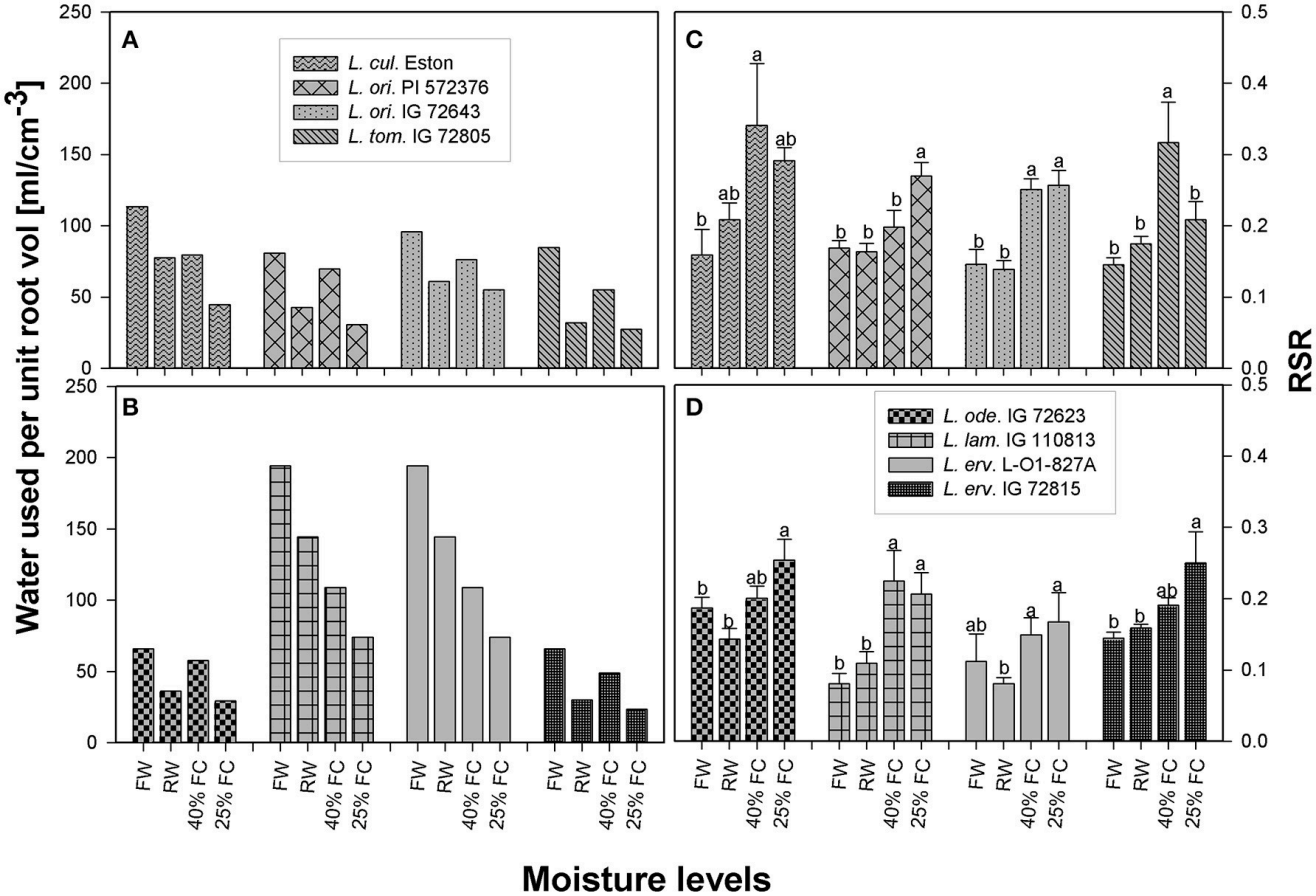
Comparison of the average amount of water transpired per unit volume **(A–B)** and root: shoot ratio **(C–D)** between cultivated and wild lentil genotypes and within wild lentil genotypes. Plants were grown under four conditions: fully watered (FW), allowed to dry to 40% of field capacity (FC) and then re-watered (RW), and allowed to dry to 40 or 25% FC and maintained at that level; [RSR, root:shoot ratio; bar]. Different lower case numbers denote significant differences at α = 5% between RSR at different moisture levels for a given genotype.

The average amount of water transpired per cm^3^ of root volume was calculated from 1 to 13 WAS for each genotype to determine how efficiently roots were conducting water. All genotypes used the greatest amount of soil water under fully watered conditions, with *L. lam*. IG 110813 and *L. erv*. L-01-827A extracting the most water from the soil profile (Figure 4). All genotypes belonging to the primary gene pool showed similar pattern, with plants extracting the least amount of water when grown at 25% FC. Re-watering did not encourage higher water extraction from the soil profile. A similar water extraction pattern was also observed for *L. ode*. IG 72623 and *L. erv*. IG 72815, which belong to the secondary and tertiary gene pools, respectively (Figures 4A,B).

The amount of roots produced in relation to shoots differed between genotypes even within the same gene pool in the presence of drought. For example, RSR of *L. cul*. Eston, was significantly higher for plants grown at 40% FC, while RSR of *L. ori*. IG 72643 was similar irrespective of drought severity (Figure 4C). In the secondary gene pool, *L. lam* IG 110813 and *L. ode*. IG 72623 had contrasting responses to drought, with the former having the highest RSR at 40% FC and the latter at 25% FC (Figure 4D); in the tertiary gene pool, *L. erv*. L-01-827A had the standard response with plants grown at 25% FC having the highest RSR followed by those at 40% FC, meanwhile *L. erv*. 72815 had significantly high RSR at 25% FC only (Figure 4D).

**Figure 5:**
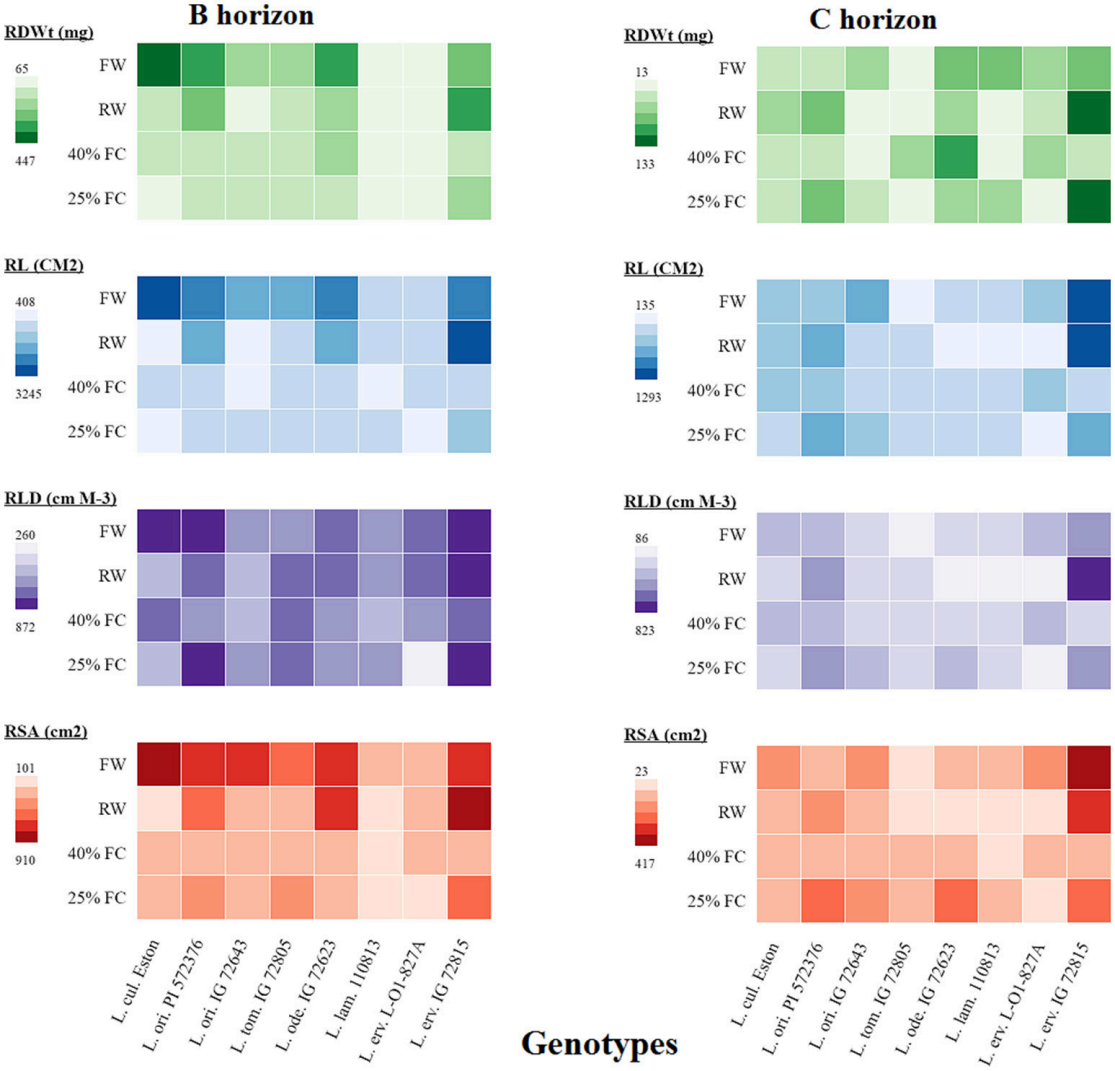
A heatmap illustrating the differences in root dry weight (RDWt), total root length (RL), root length density (RLD), and total root surface area (RSA) from the B and C soil horizons for both cultivated and wild lentil genotypes. Plants were grown under four conditions: fully watered (FW), allowed to dry to 40% of field capacity (FC) and then re-watered (RW), and allowed to dry to 40 or 25% FC and maintained at that level.

Root dry weight analysis alone provides an estimate of rooting amount but is an umbrella composed of other traits that, when analyzed further, may shade more light on root architecture. Therefore, we further analyzed root traits such as total root length (TRL), root length density (RLD), and total root surface area (TRSA) in all soil horizons and compared them to root dry weight as shown in the heatmap (Figure 5). Only the results from the B and C horizons are presented here to provide an overview of root development beyond the A horizon. In the B horizon, root biomass was lowest in *L. lam*. IG 110813 and *L. erv*. L-01-827A irrespective of the moisture level. *Lens ervoides* IG 72815 produced the most root biomass in both horizons when grown at 25% FC compared with the other genotypes. The genotypes, *L. ori* PI 572376, *L. tom*. IG 72805, and *L. ode*. IG 72623 had similar TRL at both 25 and 40% FC, meanwhile TRL was greater at 25% FC in *L. ori*. IG 72643, *L. lam* IG 110813, and *L. erv*. IG 72815. In the C horizon, *L. erv*. IG 72815 had the longest roots under fully watered, re-watered, and 25% FC compared to the other genotypes, although root length did not increase significantly when plants were grown at 40% FC. Both of the *L. ori*. genotypes produced longer roots when grown at 25% FC compared to the other genotypes. RLD followed a similar pattern to total root length, with both *L. ori*. PI 562376 and *L. erv*. IG 72815 having the highest RLD in the B horizon at 25% FC compared to the other genotypes. In the C horizon, only re-watered *L. erv*. IG 72815 plants showed a strong effect. Re-watering increased TRSA in *L. ori*. PI 572376 and *L. erv*. IG 72815 in both horizons. *Lens odemensis* IG 72623 had its highest TRSA in the B horizon when plants were re-watered and at 25% FC in the C horizon. Under fully watered conditions, *L. cul*. Eston and *L. erv*. IG 72815 had the highest TRSA in the B and C horizons, respectively meanwhile *L. erv*. IG 72815, *L. ori*. PI 572376, and *L. ode*. IG 72623 had higher TRSA when plants were grown at 25% FC in both horizons.

Further analysis with the aid of PROC MIXED procedure demonstrated that there was interaction between root traits within and between genotypes, and whether this interaction was significant or not depended on the alpha level (Table 3). Significant differences between genotypes were observed when root traits were taken into account but blocking was not significant. However, evaluation within genotypes yielded variable blocking effects. Significant differences were observed between soil horizons and genotype by horizon interaction when all traits for all genotypes were considered (Table 3). All the individual traits evaluated were significantly different between all genotypes and some interactions between traits were significant as shown in the first column of Table 3. However, to better assess root trait under different moisture levels, a different panel of traits must be selected and evaluated as shown in the rest of Table 3. We also observed that a variable number of traits would have to be considered when assessing each of the first five genotypes in Table 3 under different moisture levels compared to the last three genotypes.

**Table 3:** Summary of the effects of blocking, soil horizon, and root parameters and their interactions for all lentil genotypes (first column); comparison of same parameters within the same genotype grown at different moisture levels (remainder of columns).

**Source**	**Genotypes**								
	**All geno**	***L. cul*.**	***L. ori*.**	***L. ori*.**	***L. tom*.**	***L. ode***.	***L. lam***.	***L. erv***.	***L. erv***.
		**Eston**	**IG 72643**	**PI 572376**	**IG 72805**	**IG 72623**	**IG 110813**	**LO1 827A**	**IG 72815**
Genotypes	***	***	***	***	***	***	ns	***	***
Block	ns	*	**	***	**	ns	ns	*	ns
Horizon	***	***	***	***	***	***	***	***	***
G × H	***	–	–	–	–	–	–	–	–
RL	***	***	***	***	***	***	ns	ns	**
RSA	*	ns	ns	***	ns	***	ns	ns	ns
RAD	*	ns	ns	ns	ns	***	ns	ns	ns
RLD	**	ns	ns	ns	**	***	**	ns	ns
RV	***	**	***	***	ns	***	ns	**	ns
RL × RSA	ns	ns	***	ns	***	***	ns	ns	*
RL × RAD	***	***	ns	ns	***	***	ns	ns	ns
RL × RLD	ns	***	ns	**	*	ns	ns	ns	ns
RL × RV	**	ns	***	ns	ns	***	ns	***	ns
RSA × RLD	ns	ns	ns	ns	***	ns	ns	ns	ns
RSA × RV	ns	ns	***	ns	ns	ns	ns	ns	ns
RAD × RLD	ns	***	***	ns	**	***	ns	ns	ns
RAD × RV	***	***	ns	ns	ns	ns	ns	ns	ns
RLD × RV	ns	***	ns	**	ns	ns	ns	ns	ns
RL × RSA × RAD	***	***	ns	**	*	ns	ns	ns	ns
RL × RSA × RLD	ns	ns	ns	**	ns	ns	ns	**	ns
RSA × RAD × RLD	ns	ns	ns	*	ns	**	ns	ns	ns
RSA × RAD × RV	ns	ns	ns	**	ns	ns	ns	ns	ns
RSA × RLD × RV	**	ns	**	ns	ns	ns	ns	ns	ns
RAD × RLD × RV	ns	ns	ns	ns	***	***	ns	ns	ns

*, **, *** *Significance at alpha equals 5, 2.5, and 1% respectively; ns: not significant. [RL, Total Root length (cm); RSA, root surface area (cm^2^); RAD, root average diameter (mm); RLD, root length density (cm/m^3^); RV, root volume (cm^3^); G × H, genotype by horizon]; Geno denotes Genotypes. Moisture levels were fully watered conditions, re-watered plants, and plants maintained at 40 and 25% of field capacity after 8 weeks after sowing*.

## Discussion

Soil moisture content significantly affected the growth and development of both wild and cultivated lentil genotypes. For both situations, the number of DTF differed in response to reduced water conditions. Most genotypes grown under fully watered conditions flowered around 42 days, but there was no clear pattern under drought conditions (Table 2). Most of the parameters presented in Table 2 were within the range of that reported by Singh et al. (2014), although their results were based on a field experiment while ours was indoors. It is known that plants react to moisture deficit by either shortening or prolonging the completion of their life cycle (Turner et al., 2001). Therefore, there is need to breed for both early and late maturing lentil varieties given the forecasted increased occurrence of variable spatial distribution of rainfall and increased frequency of droughts. Early flowering would be advantageous when drought occurs after a period with sufficient available soil moisture. This work showed that in *L. cul*. Eston, severe drought provoked early flowering, a response that was not observed in any of the wild genotypes. This implies that cultivated lentil attempts to complete its life cycle as soon as possible, resulting in decreased number of seeds. Given the shallow root system of cultivated lentil, in the absence of top soil moisture, there is a greater risk of yield failure in the case of prolonged drought. Wild lentil genotypes, on the other hand, either showed either no effects or delayed flowering, a behavior that could be linked to the day length at their center of origin since the amount and quality of light has been shown to be an important factor in lentil growth (Yuan et al., 2017). Delayed flowering may be a survival strategy that ensures that enough water is available before these wild genotypes complete their life cycle. In the presence of deep root systems such as those of *L. erv*. IG 72815 and *L. ode*. IG 72623, delayed flowering allows plants to continue to continue growth since they can assess water from deeper soil horizons. However, in prolonged drought, these genotypes may fail to flower, or flowers may later be aborted since most droughts are associated with heat stress from increased temperature. From the standpoint of genetic improvement efforts, superior genotypes may be those that flower early and possess deep root systems, ensuring that under conditions of unpredictable soil moisture, the life cycle can be rapidly completed. Genotypes in the same gene pool reacted differently under both well-watered and drought conditions, implying that their genetic similarity alone cannot explain their response to drought. Rather, the interaction of their genes and the environment over time at their centers of origin may provide clues regarding the observed behaviors. For example: both *L. ode*. IG 72623 and *L. lam*. IG 110813 belong to the secondary gene pool but the DTF under all moisture levels differed significantly. *Lens odemensis* IG 72623 had a significant flowering delay (5 weeks) when plants were grown under drought conditions while *L. lam*. IG 110813 delayed flowering by only a week under the same conditions. Also, the length of time it took for some genotypes to flower was unchanged for both *L. ori*. PI 572376 and *L. tom*. IG 72805, irrespective of moisture levels. This implies that the genes controlling flowering in these genotypes may not be influenced by soil moisture level. Furthermore, based on genotyping-by-sequencing classification, *L. ori*. PI 572376 was genetically placed between *L. cul*. Eston and *L. ori*. IG 72623 (Wong et al., 2015), but its flowering response was comparatively different implying that flowering may be influenced by other factors.

Reduction in biomass was observed in both wild and cultivated genotypes in the presence of drought. This reduction could have resulted not only from moisture deficits but also from limited nitrogen fixation resulting from the reduction in both nodule number and size as reported by Zahran (1999). High biomass production is almost always associated to higher yield. However, this study showed that under drought conditions, this is not always the case. For example: *L. erv*. IG 72815 produced significantly higher biomass and RGR, but failed to produce seeds under drought conditions; re-watering only resulted in more biomass and not seeds. This implies that in the event of rain occurring after a drought spell, plant rejuvenation would be maximum in this genotype. This pattern of response could be exploited for breeding purposes where canopy improvement is the objective. On the other hand, *L. ori*. PI 572376, which had a well-developed root system produced a large amount of biomass with significantly low RGR (a very short plant), and produced high numbers of seeds. *Lens culinaris* Eston had the largest seeds even under drought while the smallest seeds were found in both *L. ervoides* genotypes. However, the number of seeds reported should not be considered absolute because lentil plants have an indeterminate growth habit. So, the termination of this experiment might have been outside the window where some of the genotypes, especially those that showed delayed flowering, might have yet to flower and produce seeds. The production of numerous small seeds may become an important factor in breeding programs when these wild genotypes are crossed with cultivated lentil given that reduced seed size may result in depressed yield (Tullu et al., 2013). This study also showed that genotypes with high biomass such as *L. ori*. PI 572376, *L. ode*. IG 72623, and *L. erv*. IG 72815, transpired less water from the soil profile, implying that they used water more efficiently compared to both the cultivated lentils and other wild genotypes.

Another strategy that plants use to evade moisture deficit is by reducing their transpiration rate, employing either morphological or physiological mechanisms (Kramer, 1980; Turner et al., 2001). All genotypes transpired the most water under fully watered conditions since water was freely available. However, most wild genotypes used water more efficiently than their cultivated counterparts. For example both *L. cul*. Eston and *L. ode*. IG 72623 produced similar amounts of biomass under fully watered conditions (Figure 2E), but *L. cul*. Eston transpired more water (Figures 3A,E). As drought progresses, plants are expected to further reduce transpiration rates in order to conserve the limited water they have. This behavior was only observed in the cultivated genotype and *L. erv*. L-01-827A (Figures 3A,G). Genotypes belonging to the secondary and tertiary gene pool plus *L. tom*. IG 72805 of the primary gene pool had similar transpiration rates at both 40 and 25% of field capacity. This implies that these genotypes may have developed mechanisms that are genetically associated with the ability of their cells to cope under drought stress. An example of this strategy is the presence of intense dark red pigmentation of leaves and stems observed in *L. erv*. IG 72815, but the specific role of this pigmentation requires further investigation. Also, *L. tom*. IG 72805 which evolved in a frost prone area, has increased amounts of trichomes on its leaves and stems, and is the only lentil species with tomentose pods. The physiological effects and role of these trichomes warrant further investigation.

RSR is an important indicator of drought resistance as most plants mobilize resources into their roots when drought conditions are encountered. Therefore, RSRs are expected to be highest for plants grown at 25% FC, followed by those grown at 40% FC, and then those that were re-watered, with fully watered plants having the least RSRs. Except for *L. cul*. Eston and *L. tom*. IG 72805, all wild genotypes mobilized significantly high amounts of resources into roots at 25% FC but the amount of resources mobilized at 40% FC differed between genotypes with no relationship to gene pool classification. Although, RSR provides information on how resources are re-allocated during water deficit conditions, it provides only a partial overview of drought sensitivity, not the complete picture of root architecture. The heat map (Figure 5) provides an overview of root traits in the B and C horizons, which are important moisture reservoirs under drought conditions. The presence of roots in these horizons can be attributed to deeper root systems that can access water and nutrients. *Lens culinaris* Eston produced its highest biomass in both the B and C horizons under fully watered conditions. When it was re-watered, more root biomass accumulated in the C horizon. This implies that the return of rain promotes root growth into deeper soil layers in cultivated lentil. For the wild lentil genotypes *L. lam*. IG 110813 and *L. erv*. L-01-827A, less root biomass was allocated to the B horizon and more in the C. Consequently, root traits (TRL, RLD, and TRSA) of these genotypes, preferentially increased in deeper soil layers. This may explain their ability to mine large amounts of water from the soil and in *L. erv*. L-01-827A, the ability to quickly complete its life cycle. *Lens ervoides* IG 72815 had the highest root trait values in all soil horizons, followed by *L. ori*. PI 572376 which produced a high number of seeds even under fully watered conditions. This study also demonstrated that it may not be necessary to evaluate all root traits for a given genotype, and that there were significant interactions among some but not all traits. In root studies, attention should therefore be focused on an identified subset, and not necessarily on all root traits (Table 3).

## Conclusions

Both cultivated and wild lentil genotypes demonstrated variable responses to drought. Most genotypes employed one or more strategies to survive in response to drought conditions, and different strategies were employed even for the same genotype. Drought avoidance, escape, and tolerance were identified as the main drought tolerance strategies used by both cultivated and wild lentils. *Lens culinaris* Eston escaped drought by flowering early, although it had shallow roots and less efficiently used water compared to some of its wild relatives. *Lens odemensis* IG 72623, both *L. erv*. genotypes (IG 72815 and L-01-827A), and *L. ori*. PI 572376 responded to drought by developing deep root systems and with the exception of *L. ori*. PI 572376, delayed flowering. *Lens lamottei* IG 110813, *L. ori*. IG 72643, and *L. erv*. L-01-827A also exhibited delayed flowering in response to drought but, when they did flower, resources were channeled toward seed production, which can be interpreted as another form of avoidance (Fang and Xiong, 2015). *Lens tomentosus* IG 72805 tolerated drought through reduction of its transpiration rates. This wide variation in responses to drought across the genus indicates that *Lens* wild species will become important for future development of lentil varieties given that all five wild species in this study can be hybridized with *L. culinaris*. This accessible gene pool represents an extensive genetic reservoir of potential strategies to improve lentils for drought tolerance. We also observed that genetic distance of genotypes did not greatly influence response to drought stress. Specific drought response strategy appeared to be more related to environmental factors that characterize their areas of origin, especially the average amount of precipitation during the growing season.

## Author contributions

LG, Responsible for design, execution, analysis of experiments, and preparation of manuscript with input from supervisor. Approved the final version presented and verified that all aspects of this work are correct. AV, Development of initial concepts for experimental work as part of grant proposals accepted by various research funding agencies. Supervised LG and revised manuscript for publication.

### Conflict of interest statement

The authors declare that the research was conducted in the absence of any commercial or financial relationships that could be construed as a potential conflict of interest.
